# Vertigo: An Atypical Presentation of Neurocysticercosis Successfully Treated With Albendazole

**DOI:** 10.7759/cureus.45722

**Published:** 2023-09-21

**Authors:** Elvis Mesa, Victoria Ruprecht, My Chau Nguyen, Damian Casadesus

**Affiliations:** 1 Internal Medicine, St. George's University School of Medicine, St. George's, GRD; 2 Internal Medicine, Jackson Memorial Hospital, Miami, USA; 3 Internal Medicine, Ross University School of Medicine, Bridgetown, BRB; 4 Internal Medicine, American University of the Caribbean School of Medicine, Cupecoy, SXM

**Keywords:** vertigo, seizure, epilepsy, obstructive hydrocephalus, anti-cysticercus igg, taenia solium, antihelmintic treatment, albendazole, ventricular ncc, intraventricular neurocysticercosis

## Abstract

A 20-year-old male presented to our facility with a worsening sensation of "the room spinning around" himself for the past three weeks. In the last week, he began to experience daily spells lasting for three hours each without losing consciousness. The patient had recently migrated from Central America six weeks prior to admission. On physical examination, his vital signs were within normal limits, with no focal neurological deficits. Magnetic resonance imaging (MRI) of the brain revealed a cystic-appearing lesion in the fourth ventricle with associated mass effect on the posterior aspect of the brainstem and mild periventricular edema. Laboratory studies were unremarkable except for a positive anti-cysticercus IgG antibody, which confirmed the diagnosis of neurocysticercosis.

Initially, surgery was considered, but the neurosurgeons advised medical management due to the small size of the lesion. The patient was started on albendazole 400 mg orally twice daily and dexamethasone 6 mg orally daily for 14 days. The patient responded well; his symptoms resolved by the eighth day. He was discharged home to complete his treatment and remained asymptomatic at the follow-up appointment two weeks later.

## Introduction

Cysticercosis is an infection caused by the larva form (cysticerci) of the tapeworm *Taenia solium*. The parasite may infect the central nervous system, causing neurocysticercosis (NCC). NCC is a common parasitic disease endemic to most developing countries and a considerable cause of hospital admissions due to epilepsy worldwide [1]. The disease is also an increasingly common diagnosis in industrialized countries because of increased immigration from endemic areas. Here, we document a case of a middle-aged immigrant with NCC of the fourth ventricle in the United States.

## Case presentation

A 20-year-old Hispanic male with no past medical history presented to our facility with a worsening feeling of sensation of "the room spinning around" himself for the past three weeks. The patient complained of episodes of feeling dizzy and lightheaded, associated with nausea and vomiting. In the last week, he began to experience daily episodes lasting for three hours each, which had progressively worsened. He denied loss of consciousness, head trauma, headache, seizures, and changes in hearing or vision. He denied recent fever, chills, cough, sore throat, runny nose, body aches, or fatigue. The patient had migrated from Central America about five months before admission. On physical examination, the patient was alert and oriented to person, place, and time. Vital signs were stable. There were no focal neurological deficits. Cranial nerves II through XII were intact, with normal sensation and strength in all extremities bilaterally. Deep tendon reflexes were 2+ bilaterally. Cardiopulmonary and abdominal examinations were normal.

**Figure 1:**
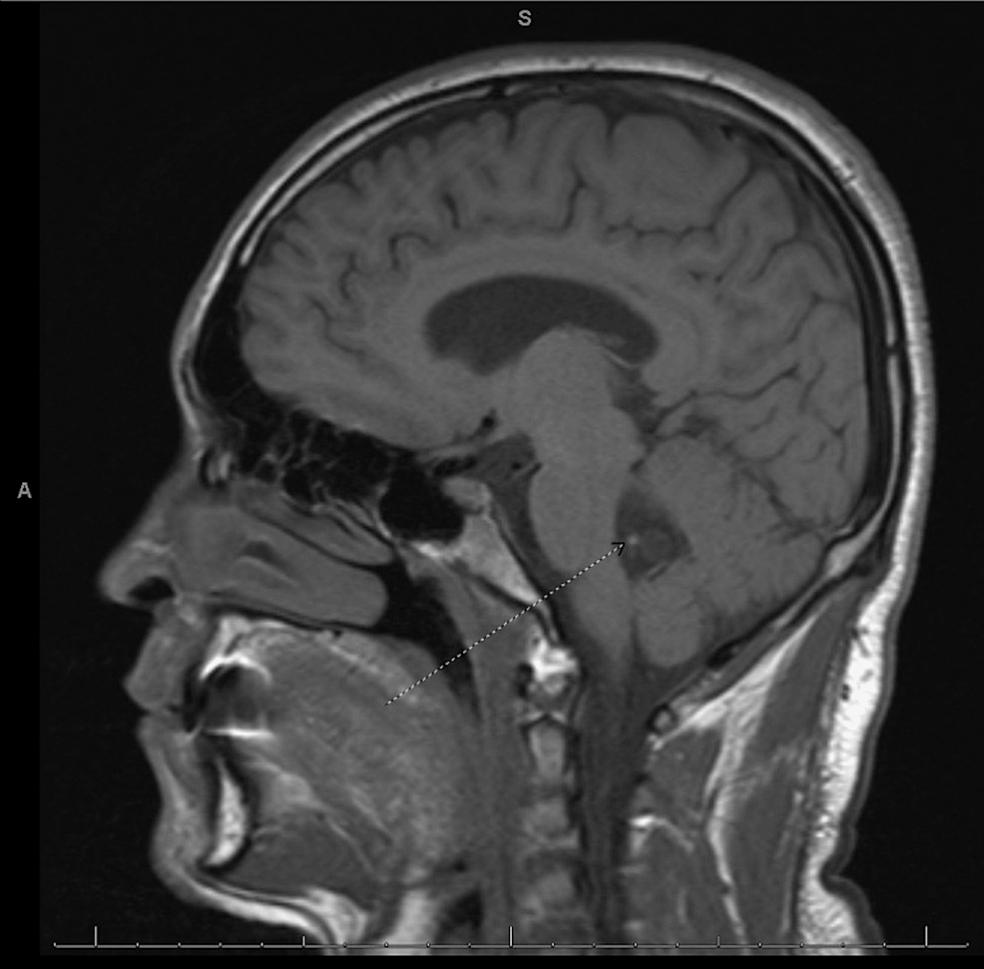
Brain MRI sagittal plane shows a cystic-appearing lesion in the fourth ventricle (arrow).

Magnetic resonance imaging (MRI) of the brain and the internal acoustic canal were obtained before and after intravenous contrast administration. Results revealed a cystic-appearing lesion in the brain's fourth ventricle with associated mass effect on the posterior aspect of the brainstem and mild periventricular edema (Figure 1).

Laboratory studies were unremarkable except for positive anti-cysticercus IgG antibody, confirming the diagnosis of NCC. Additional diagnostic tests were negative for HIV, hepatitis C, echinococcus, and Strongyloides antibodies. Liver ultrasound, spine MRI, and fundoscopic exam were negative for cysticercosis.

Initially, surgery was considered, but the neurosurgeons recommended medical management due to the small size of the lesion. The patient was started on albendazole 400 mg orally twice daily and dexamethasone 6 mg orally daily for 14 days. The patient responded well to treatment, and his symptoms resolved by the eighth day of hospitalization. He was discharged from the hospital to complete his treatment and was followed up in the outpatient clinic two weeks later. At the follow-up, the patient remained asymptomatic. A repeat MRI was recommended, but the patient refused further studies.

## Discussion

NCC is divided into parenchymal and extra parenchymal forms. Intraparenchymal NCC is the most common form of cysticercosis; it occurs in greater than 60% of cases. Extra-parenchymal NCC can occur in the ventricles, the subarachnoid space, the spine, and the eye [2]. 

The clinical picture of NCC varies from asymptomatic infection to severe, life-threatening disease. Intraventricular involvement is often more challenging to manage because it commonly presents with symptoms secondary to obstructive hydrocephalus. Commonly, symptoms develop when cysticerci become lodged in the ventricular outflow tracts, with consequent obstructive hydrocephalus and increased intracranial pressure. Associated symptoms include headache, nausea, vomiting, altered mental status, and decreased visual acuity with papilledema [3]. Occasionally, mobile cysts in the third or fourth ventricle can cause intermittent obstruction, leading to episodes of sudden loss of consciousness related to head movements (Bruns syndrome) [4].

The management of intraventricular NCC with anthelmintic medication or surgical intervention has been controversial. There are no definitively established guidelines for the anthelmintic treatment of intraventricular NCC. Nevertheless, many clinicians follow the 2013 American Academy of Neurology guidelines for treating parenchymal NCC, which recommends using albendazole and dexamethasone [5]. The potential risk of antiparasitic therapy is the exacerbation of neurologic symptoms due to inflammation around the degenerating cyst, particularly in patients with many lesions or elevated intracranial pressure. Therefore, treatment should be given under observation in a hospital with simultaneous corticosteroid administration [6].

The efficacy of anthelmintic treatment for ventricular NCC has been challenged, and a controversy still exists. Del Brutto reported a patient with intraventricular and subarachnoid locations successfully treated with albendazole [7]. Proano and coworkers reported a complete cyst disappearance in 80% of the patients treated with 16 courses of albendazole and two courses of praziquantel. In a review of 33 cases, the authors suggested that pharmacological treatment is essential to eliminate the need for surgical removal of giant cysts [8]. The presentation of intraventricular NCC with intracranial hypertension and hydrocephalus and the introduction of minimally invasive neurosurgical procedures make surgery the preferred approach for most patients with intraventricular NCC [9]. 

Initially, surgical management was considered in our patient. However, medical management was preferred due to the small single cyst and the absence of hydrocephalus. Antiparasitic therapy with anthelmintics and corticosteroids resulted in complete symptom resolution. The patient was monitored at the hospital with a repeated MRI, which showed stable findings. Our patient remained asymptomatic at his follow-up appointment but refused further MRI studies.

## Conclusions

The case highlights the success of albendazole and dexamethasone treatment of NCC of the fourth ventricle without the standard neurosurgical approach of invasive surgery. Fortunately, our patient presented with mild episodic vertigo, nausea, and vomiting without life-threatening obstructive hydrocephalus. Further case reports are needed due to limited clinical trials or clear guidelines for treating intraventricular NCC.
